# Benefits and Risks of a Staged‐Bilateral VIM Versus Unilateral VIM DBS for Essential Tremor

**DOI:** 10.1002/mdc3.13490

**Published:** 2022-06-14

**Authors:** Prarthana Prakash, Guenther Deuschl, Sarah Ozinga, Kyle T. Mitchell, Binith Cheeran, Paul S. Larson, Aristide Merola, Sergiu Groppa, Tucker Tomlinson, Jill L. Ostrem

**Affiliations:** ^1^ Department of Neurology, UCSF Weill Institute for Neurosciences, Movement Disorder and Neuromodulation Center University of California San Francisco CA USA; ^2^ Department of Neurology, Universitatsklinikum Schleswig‐Holstein, Kiel Campus Christian Albrechts University Kiel Kiel Germany; ^3^ Abbott, Clinical Research Department Plano TX USA; ^4^ Department of Neurology Duke University Durham NC USA; ^5^ Department of Neurosurgery University of Arizona Tuscon AZ USA; ^6^ Department of Neurology, Madden Center for Parkinson Disease and other Movement Disorders Ohio State University Wexner Medical Center Columbus OH USA; ^7^ Department of Neurology, Focus Program Translational Neuroscience University Medical Center of the Johannes Gutenberg‐University Mainz Mainz Germany

**Keywords:** essential tremor, thalamic deep brain stimulation, outcomes, VIM DBS

## Abstract

**Background:**

Despite over 30 years of clinical experience, high‐quality studies on the efficacy of bilateral versus unilateral deep brain stimulation (DBS) of the ventral intermediate (VIM) nucleus of the thalamus for medically refractory essential tremor (ET) remain limited.

**Objectives:**

To compare benefits and risks of bilateral versus unilateral VIM DBS using the largest ET DBS clinical trial dataset available to date.

**Methods:**

Participants from the US St. Jude/Abbott pivotal ET DBS trial who underwent staged‐bilateral VIM implantation constituted the primary cohort in this sub‐analysis. Their assessments “on” DBS at six months after second‐side VIM DBS implantation were compared to the assessments six months after unilateral implantation. Two control cohorts of participants with unilateral implantation only were also used for between‐group comparisons.

**Results:**

The primary cohort consisted of n = 38 ET patients (22M/16F; age of 65.3 ± 9.5 years). The second side VIM‐DBS resulted in a 29.6% additional improvement in the total motor CRST score (*P* < 0.001), with a 64.1% CRST improvement in the contralateral side (*P* < 0.001). An added improvement was observed in the axial tremor score (21.4%, *P* = 0.005), and CRST part B (24.8%, *P* < 0.001) score. Rate of adverse events was slightly higher after bilateral stimulation.

**Conclusions:**

In the largest ET DBS study to date, staged‐bilateral VIM DBS was a highly effective treatment for ET with bilateral implantation resulting in greater reduction in total motor tremor scores when compared to unilateral stimulation alone.

Thalamic deep brain stimulation (DBS) is an effective treatment for medication‐refractory essential tremor (ET).^1^, ^2^ This procedure is performed unilaterally or bilaterally depending on symptom severity, degree of asymmetry, and functional disability. Many patients who initially have only the most‐affected or dominant hand side treated, will eventually proceed to second‐sided surgery for better bilateral tremor control.

Despite over 30 years of clinical experience with VIM DBS in ET patients, there are no randomized controlled studies comparing VIM DBS to the best medical therapy for ET.^3^ Most studies have reported only open label short‐ and long‐term outcomes.^4^, ^5^, ^6^, ^7^, ^8^, ^9^, ^10^, ^11^, ^12^, ^13^, ^14^, ^15^, ^16^, ^17^, ^18^, ^19^, ^20^, ^21^, ^22^, ^23^, ^24^ Studies with “sufficient” data are described in a recent review.^25^ Particularly, data on bilateral VIM DBS is rare. Sixteen studies^4^, ^5^, ^6^, ^7^, ^8^, ^9^, ^10^, ^11^, ^12^, ^13^, ^14^, ^15^, ^16^, ^17^, ^18^, ^19^, ^20^ include patients who have undergone staged or simultaneous bilateral DBS implantation and only two^4^, ^5^ of these include descriptions of differences in total tremor severity and adverse event (AE) rate after a second‐sided VIM DBS when compared to unilateral treatment. The only two studies that report blinded outcomes were published over 20 years ago and are limited by small sample‐sizes. Ondo et al^4^ studied 13 subjects with ET and showed that the addition of a second‐side DBS significantly improved tremor measures contralateral to that placement without meaningful improvement on the ipsilateral side. Pahwa et al., reported similar results in nine subjects with ET.^5^ Adverse events associated with bilateral VIM DBS may include dysarthria, gait or disequilibrium problems, and paresthesias.^4^, ^5^ Studies assessing ipsilateral tremor improvement following unilateral VIM DBS implantation show minimal improvement and consistently less improvement than contralateral extremity tremor.^6^, ^7^, ^8^, ^9^, ^10^, ^11^, ^12^, ^21^, ^26^, ^27^, ^28^

A large St. Jude/Abbott funded US prospective controlled study of VIM DBS for ET using omni‐directional leads showed that unilateral and bilateral VIM DBS implantation was safe and effective in patients with medically‐refractory ET^13^ but was not designed to explore the additional benefits patients may have derived from undergoing the second side implant. Bilateral VIM DBS for ET is still considered only an “investigational” treatment according to Movement Disorders Society—Evidence Based Medicine criteria^3^ which is likely due to a deficiency of high‐quality studies. A previous sub‐analysis of this data set addressed the effects on unilateral versus bilateral VIM DBS on axial tremor outcomes.^29^ Here, we focus on overall tremor outcomes from the aforementioned pivotal trial.^13^ This sub‐analysis will provide the highest level of evidence for a staged‐bilateral VIM DBS in the treatment of ET and help document the benefits and rate of adverse events compared to single unilateral stimulation therapy.

## Methods

Detailed methods of the original 12 center prospective, controlled trial were previously published.^13^ At the beginning of the parent trial, all participants underwent unilateral VIM DBS surgery with an option for second side surgery a minimum of 6 months after initial implant. As the study progressed, there was an amendment to the protocol allowing patients to receive bilateral simultaneous VIM DBS implants upfront (for purposes of this sub analysis, we did not include these patients (n = 8)). Implantation of the constant current Libra DBS system (St. Jude Medical/Abbott Neuromodulation Division, Plano, TX, USA) was performed according to each center's standard surgical procedures. Patient's DBS stimulation settings were optimized as part of routine clinical practice. Medication therapy could be adjusted as clinically indicated throughout the study.

### Clinical Assessments

Participants were evaluated at baseline, and day 180 (±14 days) (pivotal trial's primary outcome time point). If a second lead was implanted, additional evaluations were performed at day 180 (±14 days) following the second‐sided implant. Baseline and follow‐up assessments included the Fahn and Tolosa Clinical Rating Scale for Tremor (CRST) in both the “off” and “on” DBS states (using non ‐blinded rater data), DBS parameters (i.e. active electrode, stimulation intensity, pulse width, frequency), assessment for adverse events, patient questionnaires including the Short Form‐36 Health Survey Questionnaire, Beck's Depression Inventory (BDI‐II), and Mini Mental State Exam (MMSE). CRST part A is a systematic tremor severity assessment by a rater or clinician, part B assesses handwriting (dominant hand only), drawing and pouring (each hand separately), and part C assesses functional disabilities related to speech, eating, drinking, hygiene, dressing, writing, and working. The total CRST score is a summation of CRST parts A, B, and C. CRST part C and total CRST scores were not assessed at day 180 (±14 days) following the second sided implant.

### Clinical Cohorts

The primary target cohort included patients with unilateral VIM DBS who then went on to have a staged‐bilateral implant. This cohort was used for within group comparisons to identify differences in tremor scores following unilateral and staged‐bilateral implantation. Two other control groups were defined to allow for between‐group comparisons with the primary target cohort and to aid in identifying potential confounding factors. Control cohort 2 was composed of all subjects who received a unilateral implant only (excluding the ones who later went on to have a second‐side implant). Control cohort 1 was a “matched cohort” that was comprised of a subset of subjects from control cohort 2, with unilateral implant, that were selected based on demographic and tremor severity similarity to the primary target cohort.

### Outcome Measures

The primary outcome was difference in CRST part A scores while “on” DBS at day 180 after the second‐sided VIM DBS implantation compared to the scores 180 days after the unilateral implant for the primary target cohort.

Secondary outcomes included differences in CRST sub‐scores (including lateralized tremor and midline axial tremor sub‐scores, as well as part B) at day 180 after the second‐side implant when compared to 180 days after the unilateral implant for the primary target cohort. Of note, axial tremor subscores included CRST Part A Items 1–4 and 7, all postural/rest tremors only except voice (action tremor). The differences in CRST scores and sub‐scores in control cohort 1 and control cohort 2 at 180 days post unilateral implantation respectively, were compared to the outcome measures in the primary cohort at 180 days post unilateral implantation. Patient questionnaires and MMSE scores were not included in this sub‐analysis.

Number of adverse events (device‐related and surgery‐related adverse events) were noted for the primary cohort, after unilateral and bilateral stimulation, and for the control groups. Device‐related adverse events included new or worsening cognitive and psychiatric symptoms, disequilibrium or gait impairment, dysarthria, and sensory or motor disturbances, as well as DBS system malfunction and other uncategorized side‐effects. Surgery‐related adverse events included post‐operative pain, headache, or abnormal sensations as well as surgical complications, wound healing issues, or infection.

### Statistical Analysis

A centralized electronic database contained study data from each center with automated data checks for expected value ranges and audit trails from any manual changes to the data. To create control cohort 1, pairwise matches between subjects in the primary cohort and control cohort 2 were selected to minimize the following cost function:

$$\text{Cost}=\begin{array}{l} \sum_{i=1}^{38}({\left({\mathrm{Age}}_{\text{Target}}^{i}-{\mathrm{Age}}_{\text{Control}1}^{i}\right)}^{2}+({\text{Asymmetry}}_{\text{Target}}^{i}\\ \;{-{\text{Asymmetry}}_{\text{Control}1}^{i})}^{2}) \end{array}$$

In this function, age is the subjects age in years at the time of implant, asymmetry is the absolute difference in the combined severity of the CRST for the upper and lower extremities, and cost is the total cost for a set of potential matches to the subjects in the primary cohort. Potential matches were prohibited if the subject's sex did not match or if the subject in control cohort 1 lacked data at day 180. Control cohort 2 included all subjects with unilateral‐only implants, and primary cohort included all subjects with staged bilateral implants.

Baseline demographics of age, gender, race, disease duration, disease severity, dominant handedness, and hemisphere of initial implant for the primary cohort and two control cohorts were tabulated. Total CRST scores and subscores at baseline, 180 days after unilateral VIM DBS implantation, and 180 days after second‐side VIM DBS implantation (if applicable) were tabulated as means and standard deviations for the primary cohort and two control cohorts. For each time‐point, all available data were used when computing summary statistics. Outcome measures, including differences in the CRST subscores at each given timepoint were calculated as percentages. Two sample *t* tests and paired *t* tests were performed for comparisons of baseline and changes in outcome measures within and across cohorts.

The study was approved by the United States Food and Drug Administration (FDA), and registered with clinicaltrials.gov (NCT02087046). All sites received Institutional Review Board approval prior to consenting patients. Written informed consent was obtained prior to study procedures and device implantation. Abbott (formerly St Jude Medical) sponsored the original trial.^13^ The current sub‐analysis was conducted with in collaboration with Abbott. Abbott provided the raw clinical data and re‐analysis was performed by the Abbott clinical science team under the direction of non‐Abbott investigators. Funding, interpretation and manuscript preparation were conducted by the investigators independent of Abbott.

## Results

**FIG 1:**
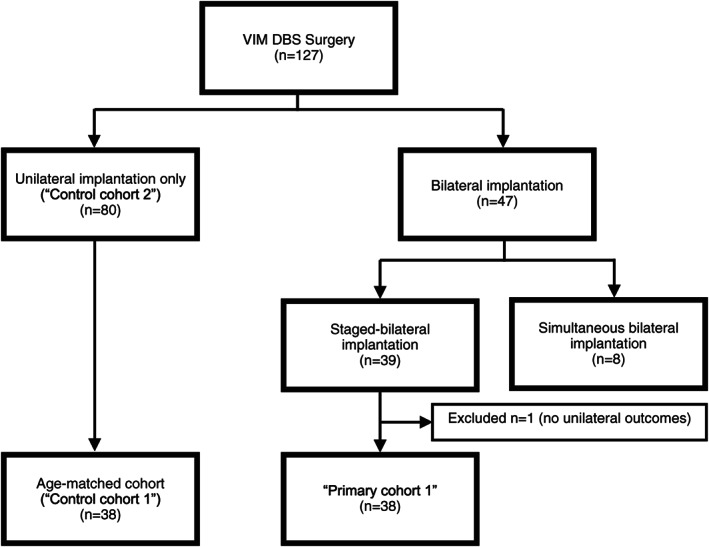
Consort diagram summarizing the categorization of participants for this sub‐analysis.

In total, 38 patients were included in the primary cohort (i.e participants with a unilateral implant who then went on to have the staged‐bilateral implant). Control cohort 1 included 38 matched unilaterally only implanted patients and the control cohort 2 included 80 patients with unilateral only implantation (Fig. 1).

Demographic data in the primary cohort group included mean age at implantation of 65.3 ± 9.5 years, mean duration of symptoms since diagnosis of 17.0 ± 11.9 years, mean duration since tremor onset of 28.6 ± 16.0 years, and baseline total Motor CRST of 17.9 ± 5.4 (Table 1). Clinical data including CRST scores and subscores at baseline, after unilateral and bilateral implantation *(if applicable)* are tabulated as means and standard deviations in Table 2.

**TABLE 1:** Patient demographics and characteristics across cohorts

Category	Primary cohort with staged bilateral DBS (N = 38)	Control cohort 1‐ matched only unilateral DBS cohort (N = 38)	*P* value	Control cohort 2‐ full cohort unilateral DBS cohort (N = 80)	*P* value
Age (yr)	65.3 ± 9.5	65.4 ± 9.7		65.8 ± 9.2	0.97
*Sex*					
Female	16 (42%)	16 (42%)	1.00	36 (45%)	0.84
Male	22 (58%)	22 (58%)		44 (55%)	
*Race or ethnicity*					
Black	0 (0%)	1 (3%)		1 (1%)	0.24
Hispanic	2 (5%)	0 (0%)		0 (0%)	
White	36 (95%)	37 (97%)		79 (99%)	
Height (cm)	172.7 ± 10.4	172.1 ± 10.1	0.69	171 ± 10	0.63
Weight (kg)	89.7 ± 18.0	84.4 ± 23.0	0.26	84 ± 22	0.19
Years since tremor onset	28.6 ± 16.0	31.3 ± 18.8	0.50	30.4 ± 18.3	0.61
Years since diagnosis	17.0 ± 11.9	16.6 ± 13.9	0.89	14.5 ± 12.0	0.28
Degree of tremor asymmetry*	1.63 ± 1.38	1.58 ± 1.15		1.90 ± 1.87	0.26
*Handedness*					
Right	32 (84%)	33 (87%)		69 (86%)	0.78
Left	6 (16%)	5 (13%)		11 (14%)	
*Most symptomatic side*					
Right	18 (47%)	25 (66%)		43 (53%)	0.65
Left	11 (29%)	7 (18%)		17 (21%)	
Equal	9 (24%)	6 (16%)		20 (25%)	
*Hemisphere first implanted*					
Right	5 (13%)	6 (16%)		13 (16%)	0.79
Left	33 (87%)	32 (84%)		67 (84%)	

* Degree of tremor asymmetry reported using absolute value of total difference between Q5 + Q8 an Q6 + Q9 on the Fahn‐Tolosa‐Marin Clinical Rating Scale for tremor (CRST). More symptomatic side determined by comparing total of Q5 + Q8 vs. Q6 + Q9.

**TABLE 2:** Outcome measures across cohorts (mean (SD))

Assessments	Primary outcome	Secondary outcomes							
	Total motor CRST score	Initial target limb CRST score	Second limb CRST score	Axial tremor score	Voice tremor score	Head tremor score	CRST part B score	CRST part C score	CRST total score
*Primary cohort*									
Baseline score^a^	17.9 ± 5.4	7.3 ± 2.2	6.4 ± 2.4	4.2 ± 2.8	1.2 ± 1.0	1.9 ± 1.6	23.8 ± 6.4	16.5 ± 4.1	58.2 ± 12.8
Day 180 after unilateral DBS^b^	8.6 ± 3.8	1.1 ± 1.3	5.9 ± 2.2	1.5 ± 1.7	0.6 ± 0.8	0.6 ± 0.8	13.5 ± 4.9	4.9 ± 4.0	26.9 ± 10.7
Change score^c^ *P* value^a−b^	9.4 ± 4.6 *P* < 0.001	6.2 ± 2.4 *P* < 0.001	0.5 ± 2.0 *P* = 0.12	2.7 ± 2.0 *P* < 0.001	0.6 ± 0.8 *P* < 0.001	1.3 ± 1.4 *P* < 0.001	10.3 ± 5.5 *P* < 0.001	11.7 ± 4.5 *P* < 0.001	31.3 ± 11.0 *P* < 0.001
**Day 180 after staged bilateral DBS^d^	3.3 ± 3.4	0.9 ± 1.1	1.8 ± 2.1	0.6 ± 1.1	0.3 ± 0.6	0.2 ± 0.6	7.6 ± 4.6	N/A*	N/A*
Change score^e^ *P* value	14.5 ± 5.7^a−d^ *P* < 0.001^a−d^ 5.3 ± 4.2^b−d^ *P* < 0.001^b−d^	6.4 ± 2.4^a−d^ *P* < 0.001^a−d^ 0.2 ± 1.4^b−d^ *P* = 0.400^b−d^	4.6 ± 2.5^a−d^ *P* < 0.001^a−d^ 4.2 ± 2.4^b−d^ *P* < 0.001^b−d^	3.5 ± 2.6^a−d^ *P* < 0.001^a−d^ 0.9 ± 1.7^b−d^ *P* = 0.005^b−d^	0.9 ± 0.8^a−d^ *P* < 0.001^a−d^ 0.3 ± 0.9^b−d^ *P* = 0.067^b−d^	1.7 ± 1.6^a−d^ *P* < 0.001^a−d^ 0.4 ± 0.9^b−d^ *P* = 0.026^b−d^	16.0 ± 6.6^a−d^ *P* < 0.001^a−d^ 5.5 ± 4.2^b−d^ *P* < 0.001^b−d^	N/A*	N/A*
*Control cohort 1*									
Baseline age matched unilateral DBS^f^	16.0 ± 5.5	6.8 ± 2.0	5.7 ± 2.4	3.6 ± 3.0	1.3 ± 1.0	1.4 ± 1.6	21.9 ± 6.6	16.5 ± 3.9	54.6 ± 12.9
Day 180 age matched unilateral DBS^g^	7.1 ± 3.8	1.2 ± 1.2	4.5 ± 2.3	1.4 ± 1.8	0.5 ± 0.7	0.4 ± 0.9	12.2 ± 6.6	5.2 ± 4.4	24.0 ± 12.7
Change score^h^ *P* value	9.1 ± 7.0^f−g^ *P* < 0.001^f−g^ 3.7 ± 0.9^g−d^ *P* < 0.001^g−d^	5.6 ± 2.2^f−g^ *P* < 0.001^f−g^ 0.3 ± 0.3^g−d^ *P* = 0.278^g−d^	1.2 ± 3.6^f−g^ *P* = 0.05^f−g^ 2.7 ± 0.5^g−d^ *P* < 0.001^g−d^	2.2 ± 3.4^f−g^ *P* < 0.001^f−g^ 0.8 ± 0.4^g−d^ *P* = 0.035^g−d^	0.8 ± 1.3^f−g^ *P* < 0.001^f−g^ 0.2 ± 0.2^g−d^ *P* = 0.154^g−d^	1.0 ± 1.8^f−g^ *P* = 0.001^f−g^ 0.2 ± 0.2^g−d^ *P* = 0.318^g−d^	9.7 ± 9.4^f−g^ *P* < 0.001^f−g^ 4.6 ± 1.3^g−d^ *P* = 0.001^g−d^	11.4 ± 6.2^f−g^ *P* < 0.001^f−g^	31.2 ± 18.8^f−g^ *P* < 0.001^f−g^
*Control cohort 2*									
Baseline FULL cohort unilateral DBS^i^	16.6 ± 6.3	7.0 ± 2.2	5.6 ± 2.7	4.0 ± 3.6	1.2 ± 1.0	1.5 ± 1.6	21.4 ± 6.8	16.4 ± 3.9	54.4 ± 13.6
Day 180 FULL cohort unilateral DBS^j^	7.1 ± 4.1	1.4 ± 1.3	4.5 ± 2.4	1.3 ± 1.7	0.5 ± 0.7	0.5 ± 1.0	11.7 ± 6.5	5.3 ± 5.0	24.0 ± 13.3
Change score^k^ *P* value	9.4 ± 6.4^i−j^ *P* < 0.001^i−j^ 3.8 ± 0.7^j−d^ *P* < 0.001^j−d^	5.7 ± 2.5^i−j^ *P* < 0.001^i−j^ 0.5 ± 0.2^j−d^ *P* = 0.068^j−d^	1.2 ± 2.8^i−j^ *P* < 0.001^i−j^ 2.6 ± 0.4^j−d^ *P* < 0.001^j−d^	2.5 ± 3.8^i−j^ *P* < 0.001^i−j^ 0.7 ± 0.3^j−d^ *P* = 0.034^j−d^	0.7 ± 1.2^i−j^ *P* < 0.001^i−j^ 0.2 ± 0.1^j−d^ *P* = 0.150^j−d^	0.9 ± 1.8^i−j^ *P* < 0.001^i−j^ 0.3 ± 0.2^j−d^ *P* < 0.140^j−d^	9.5 ± 8.5^i−j^ *P* < 0.001^i−j^ 4.1 ± 1.1^j−d^ *P* = 0.001^j−d^	11.0 ± 6.6^i−j^ *P* < 0.001^i−j^	30.3 ± 17.1^i−j^ *P* < 0.001^i−j^

* CRST part C was not administered after the staged bilateral implant. Therefore, the magnitude and change of the Part C score, and total CRST are not evaluated at this timepoint.

** 35 of the 38 subjects in the primary cohort had completed on‐stimulation CRST evaluations 180 days after staged bilateral DBS implantation.

a Baseline score for the primary cohort.

b Score at day 180 after unilateral DBS (primary cohort).

c a‐b.

d Score at day 180 after staged bilateral DBS (primary cohort).

e a‐d.

f Baseline score for control cohort 1.

g Score at day 180 after unilateral DBS (control cohort 1).

h f‐g.

i Baseline score for control cohort 2.

j Score at day 180 after unilateral DBS (control cohort 2).

k i‐j.

### Primary Cohort

The total motor CRST improved by 52.0% after unilateral (*P* < 0.001) and 81.6% after bilateral VIM DBS (*P* < 0.001), with a 29.6% greater benefit after second side implantation (*P* < 0.001). (Table 2) The second‐sided surgery resulted in 64.1% improvement in the secondarily targeted contralateral limb scores (from 7.8% to 71.9%; *P* < 0.001), 2.8% non‐significant improvement on the ipsilateral arm (from 84.9% to 87.7%; *P* = 0.40), and 21.4% improvement in axial tremor scores (from 64.3% to 85.7%; *P* = 0.005). There was a 21.1% and 25.0% improvement in head (from 68.4% to 89.5%; *P* = 0.026) and voice (from 50.0% to 75.0%; *P* = 0.058) tremor scores, respectively, following second‐sided implantation.

CRST Part B scores improved by 43.3% after unilateral stimulation (*P* < 0.001) and 68.1% after bilateral VIM DBS (*P* < 0.001), with a 24.8% greater benefit after second side implantation (*P* < 0.001).

CRST Part C scores improved by 70.3% after unilateral stimulation (*P* < 0.001) and the total CRST scores improved by 53.8% post unilateral stimulation (*P* < 0.001). CRST Part C and total CRST scores were not assessed following bilateral implantation.

### Control Cohorts

No significant differences were noted in the demographic data, years since tremor onset, diagnosis, or degree of tremor asymmetry in the control cohorts when compared to the primary target cohort (Table 1). There were also no significant differences in the CRST scores or sub‐scores at 180 days post unilateral implantation. (Table 2).

Outcomes were similar for both control cohorts and the primary cohort. Control cohort 1 had a 55.6% (*P* < 0.001) improvement in their total motor CRST scores after unilateral implantation. After unilateral implantation, contralateral limb scores and ipsilateral limb scores improved by 82.4% (*P* < 0.01) and 21.1% (*P* = 0.03) respectively. Axial, voice, and head tremor scores improved by 61.1% (*P* < 0.001), 61.5% (*P* < 0.001), and 71.4% (*P* = 0.001) respectively. CRST part B, CRST part C, and total CRST scores improved by 44.3% (*P* < 0.001), 68.5% (*P* < 0.001) and 56.0% (*P* < 0.001) respectively.

Control cohort 2 had a 57.2% (*P* < 0.001) improvement in their total motor CRST scores post unilateral implantation. After unilateral implantation, patients in control cohort 2 had an 80.0% (*P* < 0.001) and 19.6% (*P* < 0.001) improvement in their contralateral target limb scores, and ipsilateral limb scores respectively. Axial, voice, and head tremor scores improved by 67.5% (*P* < 0.001), 58.3% (*P* < 0.001) and 66.7% (*P* < 0.001) respectively. CRST part B, CRST part C and Total CRST scores also improved by 45.3% (*P* < 0.001), 67.7% (*P* < 0.001) and 55.9% (*P* < 0.001) respectively.

### Adverse Events

Number of device‐ and surgery‐related adverse events are summarized in Table 3. In the primary cohort, there were 27 device and surgery‐related adverse events following unilateral DBS implantation and 50% of these resolved. After the second side was implanted, the incidence of adverse events increased by 15.7% when compared to unilateral implantation. A total of 33 new device‐ and surgery‐related adverse events were reported following the second implant and 78.8% of these resolved. Speech, balance and cognitive impairments accounted for 33.3% of the adverse events following unilateral DBS implantation and 45.5% of the adverse events following second‐sided DBS implantation. All the balance and cognitive impairments that were reported after second‐sided DBS implantation resolved subsequently. More than half the reported speech impairments following bilateral DBS were noted to resolve as well.

**TABLE 3:** Number of device and surgery related adverse events

Adverse event (AE)	Primary cohort (n = 38)						Control cohort 1 (n = 38)			Control cohort 2 (n = 80)		
	After unilateral DBS	Resolved	*Resolved with stim change reported	After bilateral DBS	Resolved	*Resolved with stim change reported	After unilateral DBS	Resolved	*Resolved with stim change reported	After unilateral DBS	Resolved	*Resolved with stim change reported
*Device‐related*												
New or worsening cognitive and psychiatric symptoms	3	3	2	3	3	1	2	0	0	8	4	2
New or worsening disequilibrium or gait impairment	3	3	2	3	3	3	5	2	0	8	5	3
New or worsening dysarthria	3	1	1	9	5	4	2	0	0	5	0	0
New or worsening sensory or motor disturbance	5	1	1	5	5	2	6	4	3	12	6	3
DBS system malfunction	0	0	0	2	2	0	5	5	1	7	6	1
Other	3	1	1	1	0	0	0	0	0	2	1	1
*Surgery‐related*												
Post‐operative pain, headache, or jolting sensation	8	8	1	8	6	2	11	7	1	20	12	3
Surgical complication, wound healing, or infection	2	2	0	2	2	0	2	2	0	6	6	0
Total (** %)	27 (71.1%)	19 (50.0%)	8 (21.0%)	33 (86.8%)	26 (68.4%)	12 (31.6%)	33 (86.8%)	20 (52.6%)	5 (13.2%)	68 (85%)	40 (50%)	13 (16.3%)

* This refers to AE which resolved and where there was a documentation of a stimulation (stim) change. Please note that “resolved with stim change reported” does not confirm that the stimulation change was the cause for the resolution of the AE.

** Percent is calculated by dividing the total number of AE in each column by the total number of patients in the cohort and then multiplying this number with 100 to obtain a percentage.

In control cohort 1, there were 33 device‐ and surgery‐related adverse events following unilateral DBS implantation and 60.6% of these resolved at subsequent DBS programming visits. Speech, balance, and cognitive impairments accounted for 27.3% of the reported adverse events.

In control cohort 2, there were 68 device‐ and surgery‐related adverse events following unilateral DBS implantation and 58.8% of these resolved. Speech, balance, and cognitive impairments accounted for 29.4% of the reported adverse events.

There were four SAEs following unilateral DBS implantation and an additional four after the second‐sided implant. Seven of these eight SAEs were reported as unrelated to the DBS system. There were 12 SAEs in control cohort 1 and three of the 12 SAEs were reported as unrelated to the DBS system. There were 25 SAEs in the control cohort 2 and nine of the 25 SAE were reported as unrelated to the DBS system.

## Discussion

The recent MDS—Evidence Based Review^3^ recommends unilateral VIM DBS as *possibly useful* surgical options in the treatment of ET as each of them are supported by data from at least a single randomized controlled trial. On the other hand, despite its continued usage in clinical practice, bilateral VIM DBS was noted to have insufficient scientific evidence, with only two prior studies^4^, ^5^ reporting benefits of a second‐sided DBS implant when compared to unilateral implants. Both these studies reported significant improvements following a second‐sided implant; however, they were limited by small sample sizes.

The data from this re‐analysis provides further scientific evidence that second‐sided VIM DBS implantation significantly improves tremor severity when compared to a unilateral implant. Our study is the largest study to date assessing the added benefit of a second‐side DBS implant. Improvements were noted in motor CRSTs, secondarily targeted limb CRST, handwriting and pouring scores 6 months following a second‐sided implant when compared to 6 months after a unilateral implant. Changes in CRST part C and total CRST scores were not assessed following bilateral implantation. We speculate the possibility of a lack of improvement in participant reported functional disability due to the fact that the hemisphere affecting the dominant hand was implanted initially in most patients, and improvement in dominant hand function contributed most to improved function. A small study by Huss and colleagues,^7^ found no significant differences in quality of life between bilateral and unilaterally implanted patients despite greater improvements in overall tremor scores in the bilateral group. Our sub‐analysis did not include a quality‐of‐life questionnaire, and we suggest future studies investigate this interesting and non‐intuitive discrepancy between overall tremor improvement and quality of life.

Adverse events and SAEs were similar to those reported in DBS trials. The cohort of bilaterally operated patients did not have higher incidences of AE than the larger unilaterally implanted control group (i.e. control cohort 2). There was a difference in surgically induced AEs following bilateral when compared to unilateral surgeries, but not when comparing with control cohort 2, although they had two surgeries. Interestingly, the SAE rate was notably lower in the primary cohort when compared to either of the control cohorts, especially when considering only the SAEs reported as related to the system. This is despite the longer period of follow‐up for the patients in the primary cohort. We did not have a large enough bilaterally operated control group to compare the AE/SAE of one‐time versus staged surgery. In clinical practice, the number of surgically‐related AE may be smaller when bilateral procedures are done in a single‐ compared to a double‐step procedure.

Other strengths of this sub‐study include low dropout rates and comparisons with two control groups. Weaknesses include this was a post‐hoc analysis in that statistical significances were not adjusted for multiple comparisons. Also, there was a lack of racial diversity (97.5% Caucasian), and the original clinical trial was not designed to assess the added benefit of a staged‐bilateral implantation when compared to unilateral implantation and unfortunately quality‐of‐life measures were not available. We also acknowledge with newer DBS systems now available with features including segmented leads and broader parameters, adverse events in clinical practice may be less that what we have reported in these patients treated with a legacy system.

## Conclusions

These data provide further evidence that second‐sided VIM DBS implantation significantly improves tremor severity when compared to a unilateral implant providing better bilateral hand tremor control in patients with ET. Patients should be educated about the benefits of having the second side implanted to provide greater overall tremor reduction, and that there is potential increased risk of adverse events.

## Author Roles

1. Research project: A. Conception, B. Organization, C. Execution; 2. Statistical Analysis: A. Design, B. Execution, C. Review and Critique; 3. Manuscript Preparation: A. Writing of the first draft, B. Review and Critique.

PP: 1B, 1C, 2A, 2B, 2C, 3A, 3B; GD: 1A, 1B, 1C, 2A, 2C, 3B; SO: 2B, 2C, 3B; BC: 2B, 3B; PSL: 2B, 3B; AM: 2C, 3B; SG: 2C, 3B; KTM: 1A, 2C, 3B; TT: 2B, 2C, 3B; JLO: 1A, 1B, 1C, 2A, 2B, 2C, 3A, 3B.

## Disclosures

### Ethical Compliance Statement

The study was approved by the United States Food and Drug Administration (FDA), and registered with clinicaltrials.gov (NCT02087046). All sites received Institutional Review Board approval prior to consenting patients. Written informed consent was obtained prior to study procedures and device implantation. Abbott (formerly St Jude Medical) was the sponsor of the original trial.^6^ This current sub‐study was conducted with in collaboration with Abbott. Abbott provided the raw clinical data and analysis, but interpretation, funding, and manuscript preparation were conducted by the investigators independent of Abbott. We confirm that we have read the Journal's position on issues involved in ethical publication and affirm that this work is consistent with those guidelines.

### Funding Sources and Conflict of Interest

Abbott (formerly St Jude Medical) was the sponsor of the original trial.^13^ This current sub‐analysis was conducted with in collaboration with Abbott. Abbott provided the raw clinical data and re‐analysis was performed by the Abbott clinical science team under the direction of non‐Abbott investigators. No specific funding was received for this sub‐analysis. Interpretation and manuscript preparation were conducted by the investigators independent of Abbott.

### Financial Disclosures for the Previous 12 Months

PP: No additional disclosures to report.

GD: Received personal fees from Boston Scientific, Jazz, Functional Neuromodulation, Thieme publishers. He receives funding for his research from the German Research Council (SFB 1261, T1).

SO: Employee of Abbott.

BC: Employee of Abbott.

PSL: Non‐financial research support from Abbott laboratories, educational consulting for Boston Scientific and Medtronic, grants from Brain Neurotherapy Bio, Neurocrine, uniQure and Voyager, Advisory Boards for Biogen, BlueRock and Sio, consulting for Aspen, BrainXell, Corlieve, ClearPoint Neuro, Galvani, Huntington Study Group, Passage Bio, Sanofi.

KTM: Received research support from Medtronic and Deep Brain Innovations, anticipates upcoming consulting with Boston Scientific and Medtronic.

AM: Received speaker honoraria or consultancies fee from Abbott laboratories, Abbvie, and Lundbeck, and grant support from Abbvie and Lundbeck.

SG: No additional disclosures to report.

TT: Employee of Abbott.

JLO: Received consulting from Abbvie, Abbott laboratories, educational and research grants from Medtronic, Boston Scientific, Abbvie, Supernus, Amneal, Merz, Biogen, Neuroderm.
